# Author Correction: Using tyrosinase as a tri-modality reporter gene to monitor transplanted stem cells in acute myocardial infarction

**DOI:** 10.1038/s12276-025-01523-z

**Published:** 2025-08-07

**Authors:** Mei Liu, Yichun Wang, Mengting Li, Hongyan Feng, Qingyao Liu, Chunxia Qin, Yongxue Zhang, Xiaoli Lan

**Affiliations:** 1 Department of Nuclear Medicine, Union Hospital, Tongji Medical College, Huazhong University of Science and Technology, 430022 Wuhan, China; 2 Hubei Province Key Laboratory of Molecular Imaging, Union Hospital, Tongji Medical College, Huazhong University of Science and Technology, 430022 Wuhan, China

Correction to: *Experimental & Molecular Medicine* 10.1038/s12276-018-0080-7, published online 27 April 2018

After online publication of this article, the authors noticed an error in Fig. 6.

Original Fig. 6.
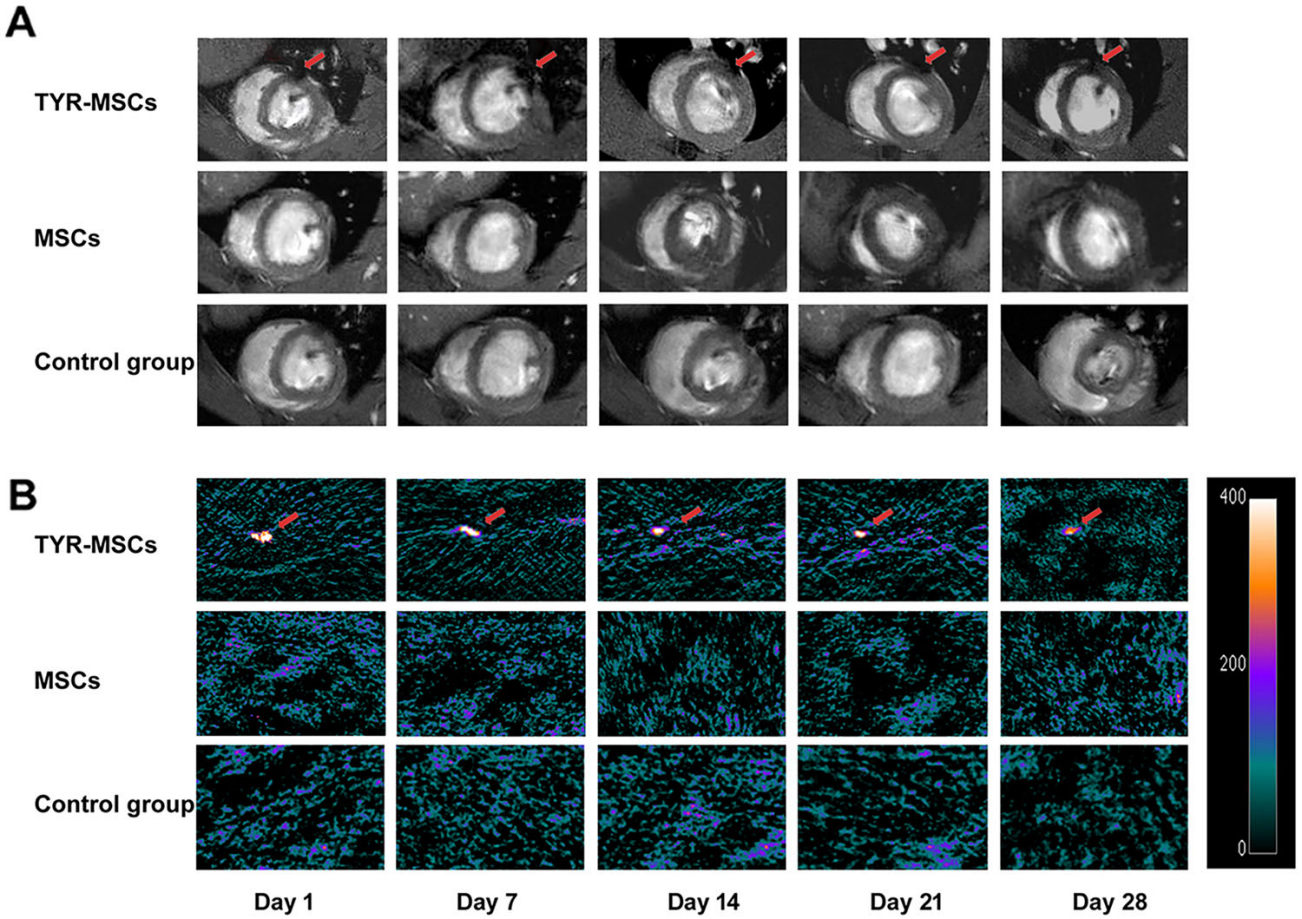


Corrected Fig. 6.
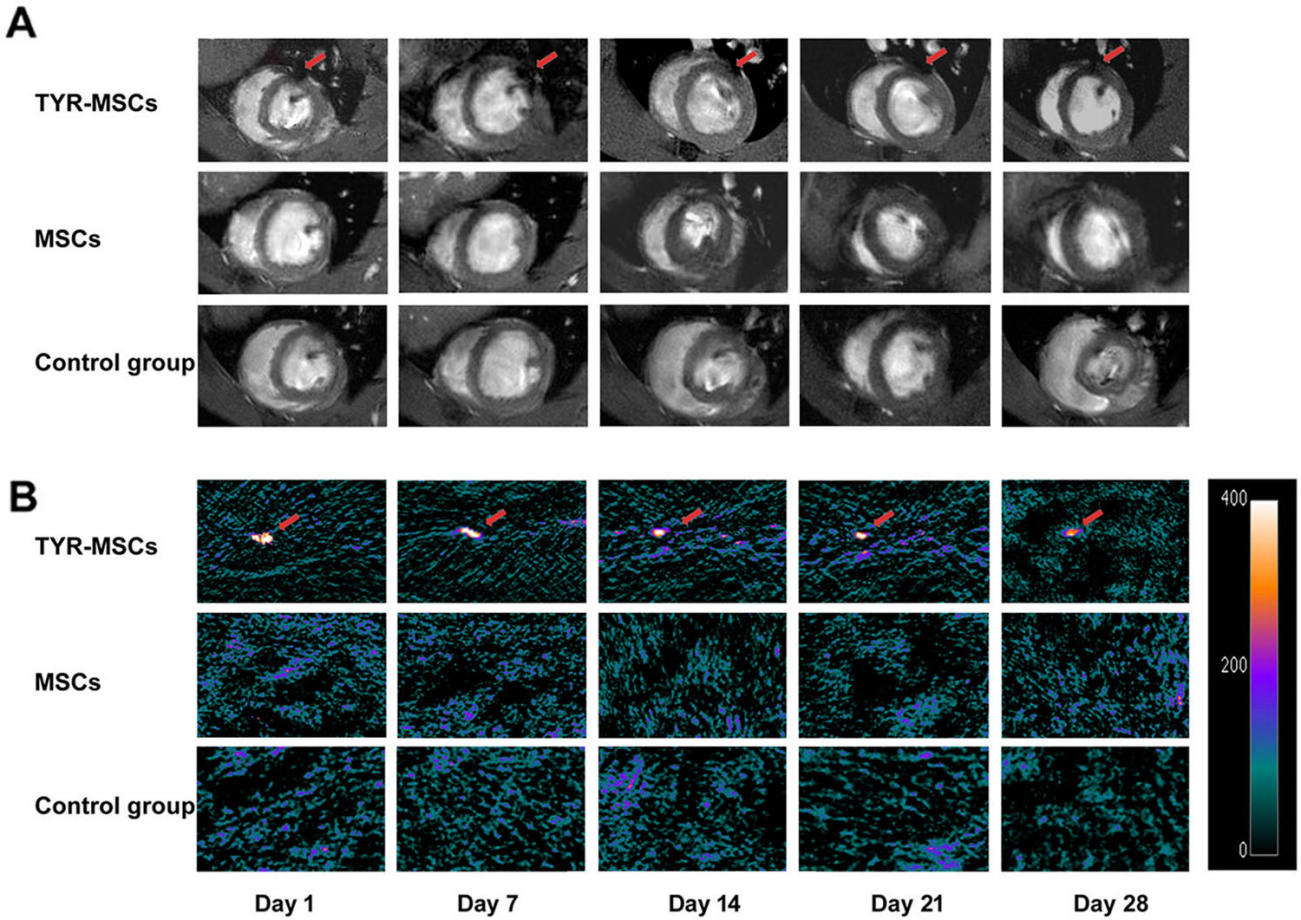


The authors apologize for any inconvenience caused.

The original article has been corrected.

